# Reactive oxygen species measure for rapid detection of infection in fluids

**DOI:** 10.1186/s13613-016-0142-8

**Published:** 2016-04-29

**Authors:** Jean Bardon, Anne-Claire Lukaszewicz, Valérie Faivre, Benjamin Huot, Didier Payen

**Affiliations:** Department of Anesthesiology and Critical Care and SAMU, Hôpital Lariboisière, AP-HP, Paris, France; Université Paris Diderot, Sorbonne Paris Cité, Paris, France; Inserm U1160, 75010 Paris, France

**Keywords:** Polymorphonuclear neutrophils, Inflammation, Infection, Critical care, Diagnosis, Bronchoalveolar lavage, Ascites, Pleural effusion, Reactive oxygen species

## Abstract

**Background:**

Early detection of infection is critical to rapidly starting effective treatment. Diagnosis can be difficult, particularly in the intensive care unit (ICU) population. Because the presence of polymorphonuclear neutrophils in tissues is the hallmark of inflammatory processes, the objective of this proof of concept study was to determine whether the measurement of reactive oxygen species (ROS) could be an efficient diagnostic tool to rapidly diagnose infections in peritoneal, pleural and bronchoalveolar lavage (BAL) fluids in ICU patients.

**Methods:**

We prospectively included all patients hospitalized in the 21-bed surgical ICU of a teaching hospital from June 2010 to February 2014 who presented with systemic inflammatory response syndrome with suspicion of a peritoneal or pleural fluid or pulmonary infection needing a BAL. Instantaneous basal ROS production was measured in fluids and after phorbol 12-myristate 13-acetate (PMA) stimulation. We compared patients with infected fluids to those with non-infected fluids.

**Results:**

The overall ICU mortality rate was 34 %. A majority of patients were sampled following a delay of 5 days (2–12) after ICU admission, with most receiving antibiotics at the time of fluid sampling (71 %). Fluids were infected in 21/65 samples: 6/17 peritoneal fluids, 8/28 pleural fluids and 7/20 BALs. ROS production was significantly higher in the infected than in the non-infected group at baseline and after PMA stimulation in the peritoneal and pleural fluids but not in BAL.

**Conclusion:**

Assessing instantaneous ROS production appears as a fast and reliable diagnostic method for detecting peritoneal and pleural fluid infection.

**Electronic supplementary material:**

The online version of this article (doi:10.1186/s13613-016-0142-8) contains supplementary material, which is available to authorized users.

## Background

Patients with an infection while in the intensive care unit (ICU) have a higher mortality rate than non-infected ICU patients [1]. Unfortunately, delayed treatment of infections in the ICU has been shown to worsen prognosis [2]. Because of this, analyses of blood samples and human fluids must be performed rapidly prior to the administration of broad-spectrum antibiotics, which are eventually adapted to the microbiological test results within the subsequent 48–72 h. The development of multidrug-resistant bacteria, first as part of the ecology of the patient population and then of the ICUs, highlights strongly the need to give antibiotics to infected patients only. Infection is difficult to diagnose when systemic inflammation is present for multiple potential reasons. In addition, the compartmentalization of infection may complicate the diagnostic strategy, necessitating a delay in the fluid sampling required to diagnose an infection. The availability of a more rapid test when infection is suspected might be useful to eliminate sterile inflammation, confirm an infection in the tested compartment, or consider the presence of infection even if direct examination was negative while awaiting culture results.

Polymorphonuclear neutrophils (PMNs) are the first line of defense against bacterial infection. In actuality, the presence of PMNs in tissues is the hallmark of inflammatory processes related to different triggers [3]. Among their activated functions, the production of reactive oxygen species (ROS), mainly from NADPH oxidase activation, is an essential step in the killing of bacteria [4]. This property can be deleterious for tissue cells, especially when inflammation is sterile. We hypothesized that PMNs’ ROS production, as we previously published [5], could be higher during infection-induced inflammation than in sterile inflammation. This could help diagnose peritoneal, pleural and bronchoalveolar fluid infection more rapidly.

## Methods

### Population

This study was approved by the Ethical Committee of the “Société de Réanimation de Langue Française” (CE SRLF 14-07), which waived the requirement of written informed consent. Information about this research protocol, however, had to be given to the patient if possible or to his/her relatives. Data were collected in the 21-bed surgical ICU of a teaching hospital from June 2010 to February 2014. Patients were screened when they had a systemic inflammatory response syndrome (SIRS) and when a peritoneal, pleural or pulmonary infection was suspected. Fluids from the peritoneum, pleural space or bronchoalveolar lavage were sampled for the diagnosis of infection according to the decision of the independent intensivist in charge.

### Definitions of infection

Peritoneal fluid infection was diagnosed in accordance with recommendations from the 2007 French Consensus Conference [6] and international guidelines [7]. These included the presence of bacteria after culture or PMN count ≥250/mm^3^ associated with local or general symptoms of inflammation. A determination of infected pleural fluid was made when the quantitative culture was positive for bacteria as defined by Light et al. [8]. Histologic findings describing the purulent sample were also used to identify pleural infection [9, 10]. Lung infection detected by BAL was defined by a bacterial quantitative culture ≥10^4^ CFU/ml [11–14], with a cell count ≥10^5^/mm^3^ containing more than 50 % PMNs [15].

### Data collection

The collected data included demographic characteristics (age, sex), reason for admission to the ICU, severity scores (Sequential Organ Failure Assessment Score: SOFA and Simplified Acute Physiology Score: SAPS II), duration of time from admission to fluid sample analysis and outcome.

Concomitant sites of infection and the antibiotic regimen, if any, were also recorded. The physician in charge decided all fluid samplings. BAL was performed using a classic protocol. Specifically, 100 ml of saline was gently injected during bronchoscopy and retrieved from the lung area of interest, before being aliquoted in three different tubes. Fluid samples were analyzed within 2 h after collection with a leukocyte and PMN count, protein level and ratio with plasma level, and the presence or not of bacteria before culture. In addition to the routine checking, ROS measurement was performed immediately on fresh samples.

### Measurements of ROS production by luminometry

ROS production was measured according to the method previously described [5, 16]. Briefly, a fluid sample (250 μl) was diluted in Hanks’ balanced salt solution (HBSS; Invitrogen, Cergy-Pontoise, France) to a final volume of 1 ml and then incubated with luminol (50 μM; Sigma) for 10 min at 37 °C in the dark. As a functional test, basal production of ROS (basal condition) was compared with a sample that had been stimulated with 10^−7^ M of phorbol 12-myristate 13-acetate (PMA; Sigma) added in sample just before luminometry allowing a dynamic approach of PMN ROS production; each condition was assayed in duplicate. Immediate analysis was conducted during a 20-min period using a luminometer (AutoLumat Plus LB 953; Berthold Technologies, Bad Wildbad, Germany). The signal was recorded as relative light units (RLUs) for a duration of 1 s every minute. The results were expressed as the area under the curve (AUC) of luminescence during the 20 min (Additional file 1: Figure S1). The time delay before having the quantitative level of ROS production averaged 35 min per patient. Considering the status of circulating cells as the reference, the ROS measurements in fluid were compared to those obtained from whole blood (40 μl) sampled at the same period of day in the same patient and to healthy volunteers’ blood. The potential variability in the reactivity of luminol was avoided by regularly preparing new luminol solutions and repeating tests over several weeks with decreasing concentrations of stable H_2_O_2_ (Chimie Recherche Environnement Evolution, Taverny, France). We repeated tests in each of seven patients’ blood samples and showed a good reproducibility of AUC calculation: Correlation test showed *R* = 0.99 and 0.90 in control and PMA conditions, respectively (*p* < 0.0001).

### Statistical analysis

Quantitative variables are expressed as the median and interquartile range, while qualitative variables are expressed as the percentage. Nonparametric Mann–Whitney, Wilcoxon’s paired test and Chi-square tests were used when appropriate, with the alpha set to 0.05. The respective role of the individual cell activity versus the number of cells on global ROS production was tested by comparing the AUC after normalization (or not) by the number of cells. We also used correlation tables using Statview^®^ (Statview^®^, SAS Institute Inc. Copyright, North Carolina, USA).

### Strategy for analysis

First, the ROS production per PMN in healthy volunteer blood was compared to the values obtained in our cohort. Within the cohort, the ROS production per PMN in blood was compared to ROS production in fluids. ROS production in fluids was then compared between infected and non-infected fluids.

## Results

**Fig. 1:**
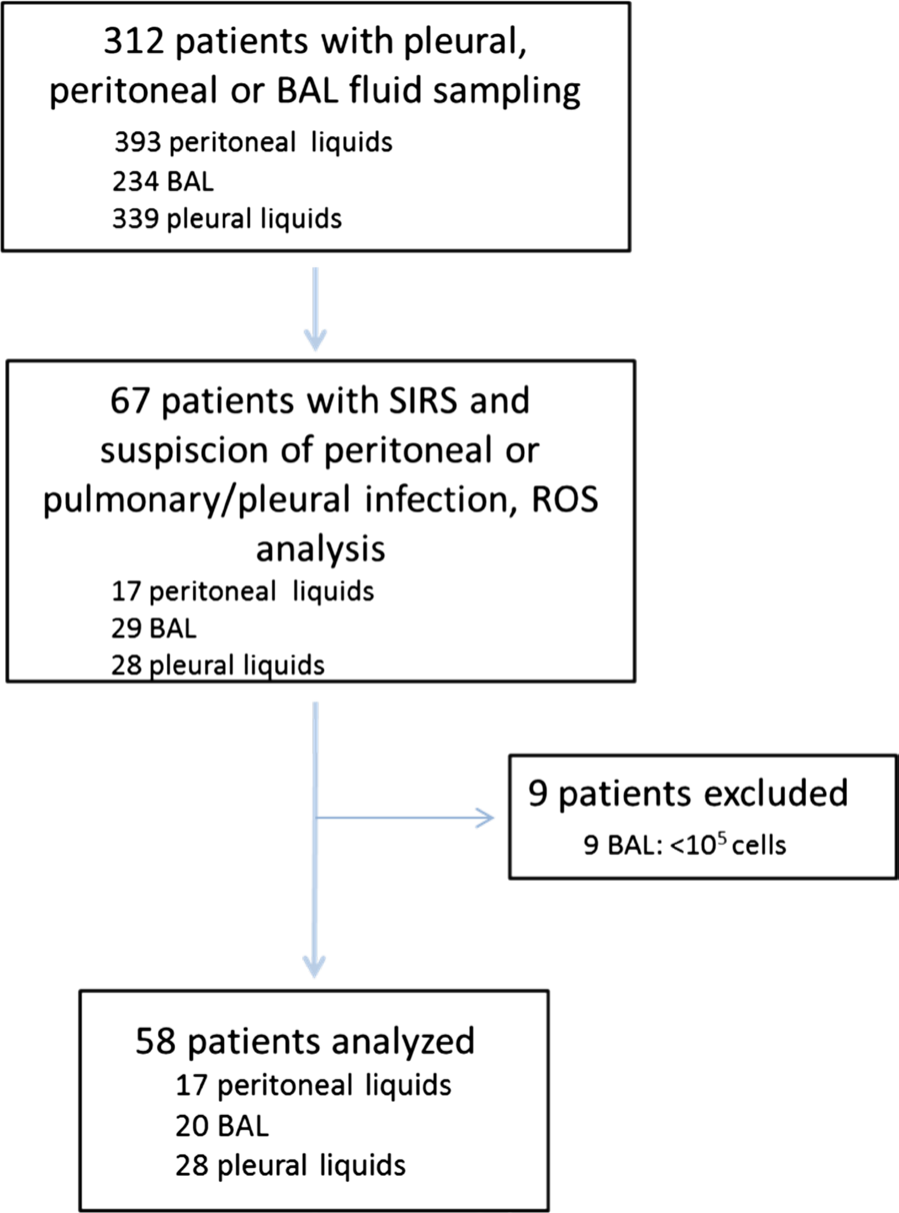
Study flow chart. 58 patients analyzed, corresponding to 17 peritoneal liquids, 28 pleural liquids and 20 bronchoalveolar lavages

During the study period, 312 patients had peritoneal, pleural or BAL fluid sampling. Among them, we analyzed 67 patients with SIRS and suspected of having an infection requiring fluid sampling that was also analyzed for ROS analysis (flow chart, Fig. 1). Nine patients with a BAL containing <10^5^ cell count/mm^3^ were excluded from the analysis. Finally, 58 patients were analyzed with 17 peritoneal fluid samples, 28 pleural fluid samples and 20 BALs. Some patients were explored at different sites. Sample characteristics are summarized in Table 1. In the group with peritoneal fluid analysis, the reason for ICU admission was mainly the presence of postoperative peritonitis, gut hemorrhage and acute respiratory failure. The pleural fluid and BAL groups essentially had acute respiratory failure, major trauma or heavy surgical procedure.

**Table 1:** Characteristics of patients according to the presence of infection in the biological sample

	Infected (*n* = 17)	Non-infected (*n* = 41)	All patients
Age (years)	58 (53–66)	59 (49–73)	59 (51–72)
Male sex	9 (53 %)	2 (59 %)	33 (57 %)
SIRS	17 (100 %)	41 (100 %)	58 (100 %)
Temperature (°C)	37.5 (37–38)	37.4 (36.6–38)	37.5 (36.6–38)
SOFA	8 (5–10)	7 (4–11)	7 (4–10)
SAPS II	51 (41–57)	41 (32–46)	43 (34–51)
ICU mortality *n* (%)	8 (47 %)	12 (29 %)	20 (34 %)
Time between ICU admission and sample analysis (days)	10 (1–20)	4 (2–9)	5 (2–12)
Blood PMN (/mm^3^)	14,904 (10,647–22,400)	10,530 (8550–17,860)	11,023 (8747–20,742)
Antibiotics at the time of sampling	14 (82 %)	27 (66 %)	41 (71 %)
Steroid treatment at the time of sampling	2 (12 %)	6 (15 %)	8 (14 %)

Quantitative variables are expressed as medians (25th–75th percentiles) and qualitative variables as frequencies (%)

*ICU* intensive care unit, *SOFA* Sequential Organ Failure Assessment Score, *SIRS* systemic inflammation response syndrome, *SAPS II* Simplified Acute Physiology Score, *PMN* polymorphonuclear neutrophils

The overall ICU mortality rate was 34 %, with 47 % of the infected group and 29 % of the non-infected group (*p* = 0.21). Fluids were infected in 21/65 fluid samples, including 6/17 peritoneal fluids (only one fluid was considered infected due to 730 PMNs/ml and no bacteria cultured), 8/28 pleural fluids and 7/20 BALs. The most frequently identified bacterium in the thoracic samples was *Pseudomonas aeruginosa* (2/8 in pleural fluid; 2/7 in BAL). Enterobacteriaceae (2/6) and *Candida* sp. (3/6) were the most frequent microorganisms in peritoneal fluid. A majority of patients (71 %) were treated with antibiotics at the time of sampling. Blood samples isolated bacteremia in three patients. The proportion of patients receiving steroid treatment was the same in the infected and non-infected groups with 12 and 15 %, respectively (*p* = 0.77).

### ROS production in blood in patients compared with healthy volunteers

**Fig. 2:**
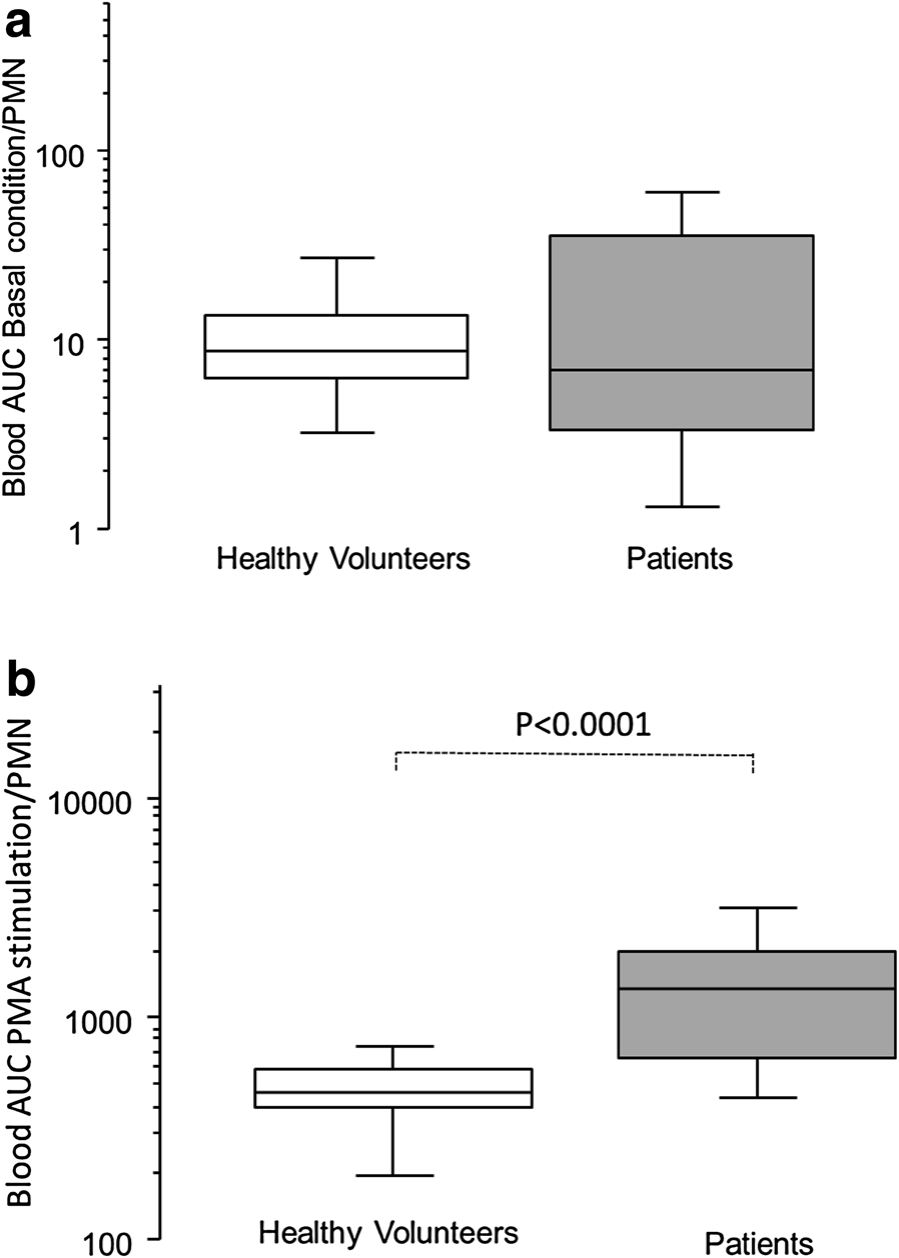
Healthy volunteers and patients’ ROS production per PMN in the blood. **a** Basal ROS production [area under the curve (AUC), logarithmic scale] per PMN in blood. Healthy volunteers: *n* = 21; patients: *n* = 49. **b** ROS production after PMA stimulation per PMN in blood. Healthy volunteers: *n* = 21; patients: *n* = 49. *Box plot* represents median and 25–75 % interquartile

Compared with healthy volunteers (HV), patients had a significantly higher leukocyte count [13,800/mm^3^ (11,175–23,800) vs 5400/mm^3^ (4500–6400), *p* < 0.0001] and a higher PMN count [11,023/mm^3^ (8747–20,742) vs 3700/mm^3^ (2510–4295), *p* < 0.0001]. While the ROS production in blood (AUC/PMN) after PMA stimulation was significantly higher in patients than in HVs, this was not observed in the basal condition (Fig. 2). As expected, such a difference was largely significant in the absence of cell normalization in the basal condition and after stimulation, potentially reflecting the “global oxidative stress” in patients’ blood, mainly due to PMNs (data not shown).

### Comparison of ROS production between blood and fluids in patients

**Fig. 3:**
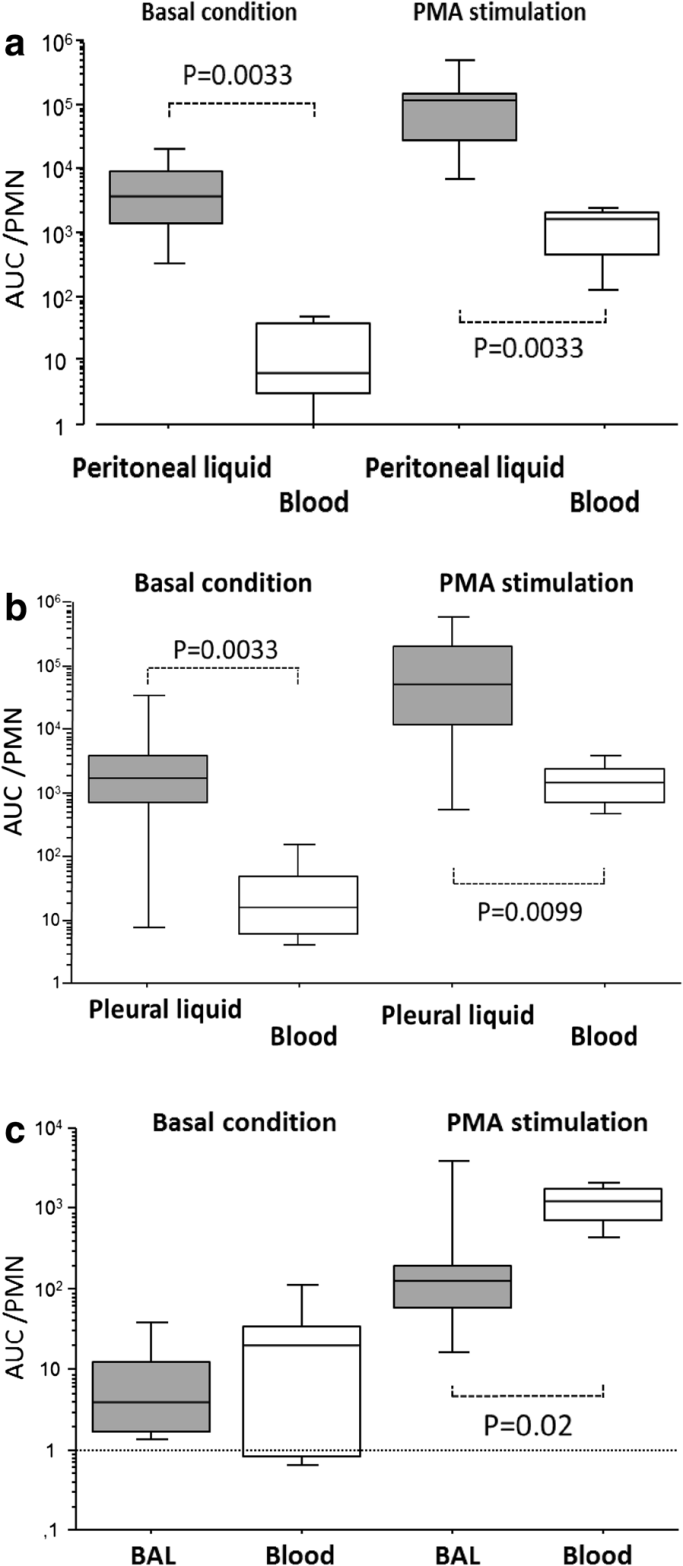
Comparison of ROS production between blood and fluids from patients. ROS production [area under the curve (AUC), logarithmic scale] per PMN in peritoneal liquid and blood (**a**, *n* = 12), pleural liquid and blood (**b**, *n* = 23) and BAL and blood (**c**, *n* = 19), under basal conditions and after PMA stimulation. *Box plot* represents median and 25–75 % interquartile

The ROS production per PMN in peritoneal fluid was significantly higher than that in blood, in both the basal and PMA conditions (Fig. 3a). Similar results were observed for pleural fluid in the basal condition and after PMA stimulation (Fig. 3b).

For BAL, no difference was observed in the basal condition. After PMA stimulation, the AUC/PMN in the BAL was lower than the AUC/PMN in blood (Fig. 3c).

Nine fluids did not have a corresponding blood sample analyzed.

### ROS production in fluids

#### Peritoneal fluid

**Fig. 4:**
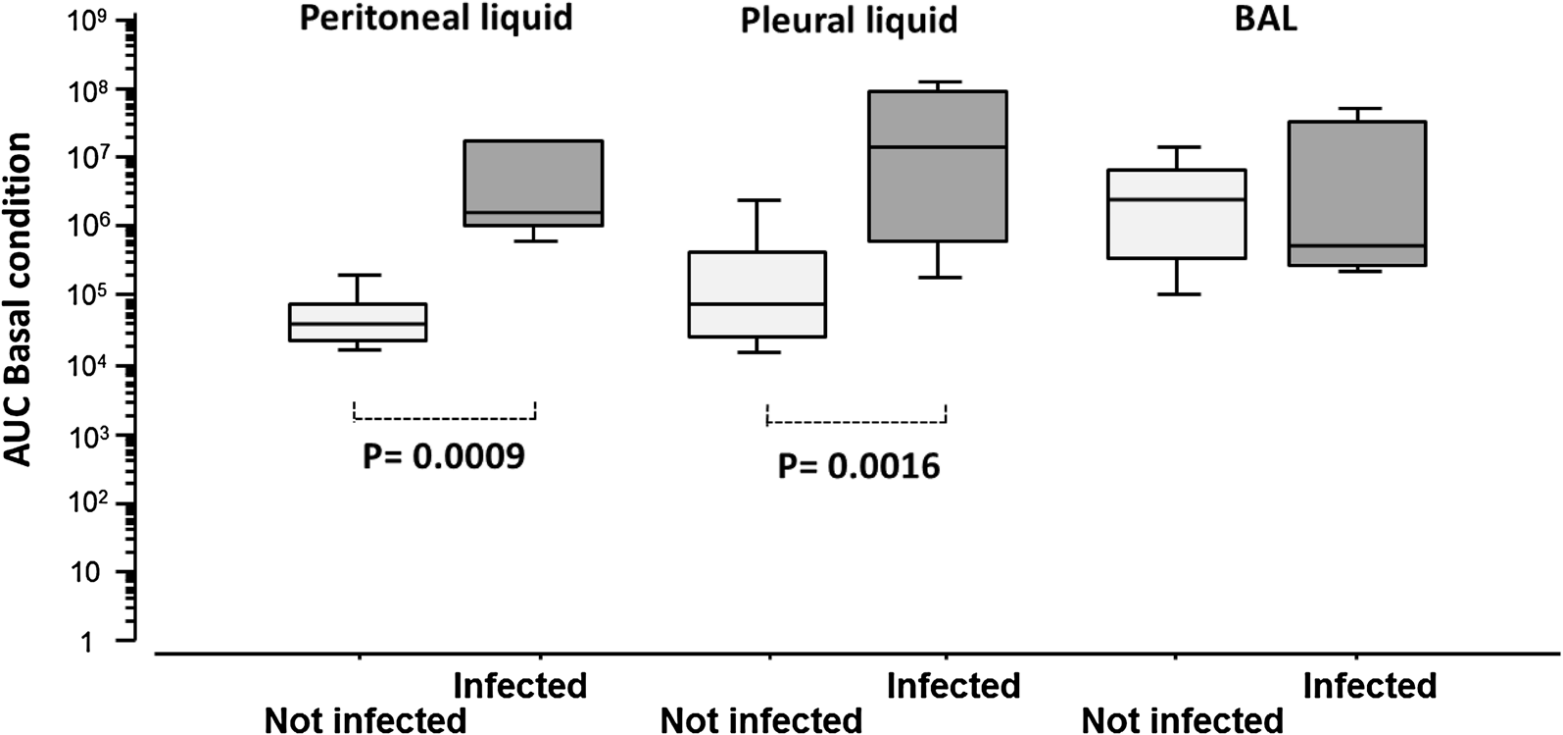
ROS production in fluids from patients according to infection. ROS production under basal conditions according to infection [area under the curve (AUC), logarithmic scale]. Number of patients with peritoneal fluid (infected/non-infected) *n* = 17 (6/11), pleural fluid *n* = 28 (8/20) and bronchoalveolar lavage (BAL) *n* = 20 (7/13). *Box plot* represents median and 25–75 % interquartile

Among the 17 peritoneal fluid samples, six (35 %) were diagnosed as infected with a median PMN count of 400/mm^3^ (361–400) versus 22/mm^3^ (4–32) in the non-infected group (*p* = 0.002). Twelve were primary peritonitis and five were secondary peritonitis. Basal ROS production was significantly higher in the infected group versus the non-infected group (Fig. 4) and after PMA stimulation (Additional file 2: Figure S2). Such a difference was not observed after normalization per PMN (data not shown). Correlation between PMN count and ROS production was 0.82 in basal condition and 0.13 with PMA (Additional file 3: Figure S3A).

#### Pleural fluid

Among the 28 pleural fluid samples, eight (29 %) were diagnosed as infected but with no difference in the PMN count from that of the non-infected group [693/mm^3^ (337–21,953) and 478/mm^3^ (121–2237), respectively (*p* = 0.4)]. The ROS production was significantly higher in the infected fluid under both the basal (Fig. 4) and stimulated conditions (Additional file 1: Figure S1). No difference was observed after normalization per PMN (data not shown). No correlation was established between PMN count and ROS production in basal condition and PMA (Additional file 3: Figure S3B).

#### Bronchoalveolar lavage

Seven (35 %) BALs among the 20 samples were diagnosed as infected. The PMN count did not differ significantly [59,250/mm^3^ (27,000–264,300) vs 118,900/mm^3^ (98,895–398,400) between the groups with or without infection (*p* = 0.43)]. ROS production in the basal condition or after PMA stimulation was not significantly different between the infected and non-infected groups (Fig. 4). No difference was observed after normalization per PMN (data not shown). No correlation was established between PMN count and ROS production in basal condition and PMA (Additional file 3: Figure S3C).

## Discussion

The results of this preliminary study observed that ROS production was higher in the infected peritoneal and pleural fluids, an effect that was not observed in BAL. Measurement of ROS production could be a potential early screening method for detecting the presence of bacteria in patients’ biological fluids.

The ICU context is challenging for infection diagnosis because a high proportion of patients receive antibiotics and exhibit systemic inflammation symptoms that can relate to many different etiologies. The studied cohort was severe, with a high median SAPS II (43) and an overall ICU mortality rate of 34 %. Fluids were sampled after a median delay of 5 days post-admission, and 71 % of these patients received antibiotics at the time of sampling; furthermore, the infected and non-infected groups did not differ in body temperature or blood PMN count; therefore, the blood leukocyte count in the present study did not help differentiate between sterile and infection-induced inflammation.

We hypothesized that the functional capability of PMNs close to the infected organ, especially the release of ROS, might help to separate these mechanisms of inflammation. If this hypothesis is valid, it may speed the diagnosis of infection because the ROS test requires only a few minutes versus hours to days for microbiological exams. The clinical decision to administer, change, or stop antibiotics could then be made more rapidly. If prescribed after screening for a positive ROS test, antibiotics could be adapted later based on the findings from microbiology scans for the type and sensitivity of the bacteria. Alternative diagnostic methods for detecting infected peritoneal and pleural liquids and BAL have been studied [17–22]. However, most of them excluded critically ill and postoperative patients and those receiving antibiotics. The present results on ROS correspond to an instantaneous production [5, 16], which differs from other techniques based on an accumulation of altered fluid components by ROS (nitrosylated and oxidated proteins) [23].

The hypothesis that the production of ROS by PMNs would be higher from fluid samples closer to the infected organ than in sterile inflammation is reasonable. PMNs are the first-line cell type in innate immunity and are activated by the presence of bacteria, with the stimulation of NADPH oxidase [4, 24–26]. NADPH oxidase is assembled at the surface of the phagosome and at the cell surface. Its activation allows the production of superoxide anion and hydrogen peroxide. This ROS production damages proteins, DNA and membranes, activates dendritic cells [27], participates in the activation of lymphocytes [28] and the production of pro-inflammatory cytokines [29] and activates the inflammasome [30].

In the present study, the ROS production per PMN in biological fluids was higher than the ROS production per PMN in the blood, except for BAL fluid. Indeed, leukocytes that infiltrate tissues are activated as they roll on the endothelial surface due to chemoattractant agents related to pathogens or damage-associated molecular patterns (DAMPs) [31]. Therefore, the difference observed in peritoneal and pleural fluid was not related to that status of PMNs in the blood circulation before infiltration.

The main result of this study is the major increase in ROS production at baseline and after PMA stimulation in peritoneal and pleural infected fluids compared with non-infected fluids in inflammatory patients. ROS were always measurable even in complex inflammatory fluids with PMN impossible to count. The difference between infected and non-infected groups was not solely due to a higher PMN count since correlations between PMN count and ROS production were low, except for peritoneal liquid tested in basal condition (Additional file 3: Figure S3A, B, C). Exposition to DAMPs, ischemia and aging are some non-infectious stimuli inducing PMN recruitment to tissues and ROS production [32–35]. Therefore, the observation of a higher amount of ROS during infection in this very specific inflammatory ICU population strengthens the observed results. Additionally, it appeared that there are distinct pathways for neutrophil attraction toward sterile or non-sterile foci. It was demonstrated that hydrogen peroxide (H_2_O_2_) and ROS formation was dispensable for leukocyte recruitment during bacterial infection and not for sterile inflammation. This may fit well with the findings of higher ROS production in infected versus non-infected tissue [3, 36]. For these reasons, we considered that ROS test would be adapted to different complex situations of ICU practice for early triage of infection source, before results of PMN count or microbiology in suspected infected fluid.

We did not observe any association regarding BAL between ROS production and the infection status. One explanation could pertain to the fact that BAL was considered infected if a culture was found to be ≥10^4^ CFU/ml [11], and then 20 % of samples had an infra-threshold bacteria culture that led to the elimination of infection rather than colonization, which might have increased ROS production. Furthermore, if macrophages predominated in non-infected BAL, they could produce ROS such as PMN and increase ROS levels in the non-infected group. Nevertheless, ROS measure does not seem suitable as a preliminary infection diagnostic tool for BAL due to the heterogeneity of its realization; it should be assessed in a specific study.

The first limit of our study was the small effective; herein we present preliminary results supporting the potential interest for early screening of different types of fluids in addition to CSF [16]. This approach would be of particular interest for the early screening of infection at patient’s admission as a complement to bacteria identification technics among which PCR and mass spectroscopy will considerably speed up infection diagnosis. Even if we were cautious for reagent quality and experiment conditions, we have to develop a standardized test before transposition to clinical practice.

Our study was limited by the small effectives with high variability of cell count in fluids for clarifying the impact of PMN count on test results. High ROS production in infected fluid might not only result from PMN number but also from ROS activity per PMN. The only correlation, between cell number and ROS production, was in peritoneal samples. This could result from the definition of infection only, because this definition was based on cell number in samples, when it was not the case for the other fluids.

## Conclusion

The measurement of instantaneous ROS production in peritoneal and pleural fluids appears to be an efficient and rapid method for diagnosing infection, and it was not suitable for BAL. This method may help speed the diagnosis of peritoneal and pleural infection, as well as decrease the cost and delay in the administration of antibiotics. These preliminary results should be confirmed in a prospective multicenter study in order to define an adequate threshold, using a standardized test.

## Additional files

10.1186/s13613-016-0142-8 Area under the curve of luminescence: HBSS and PMA.
10.1186/s13613-016-0142-8 ROS production in stimulated condition by PMA.
10.1186/s13613-016-0142-8 Correlation tables between ROS production and PMN count in biological liquids.

## Abbreviations

BAL: bronchoalveolar lavage
CFU: colony-forming units
DAMPs: damage-associated molecular patterns
HBSS: Hanks’ balanced salt solution
HV: healthy volunteers
ICU: intensive care unit
PMA: phorbol 12-myristate 13-acetate
PMNs: polymorphonuclear neutrophils
ROS: reactive oxygen species
SAPS II: Simplified Acute Physiology Score
SIRS: Systemic inflammatory response syndrome
SOFA: Sequential Organ Failure Assessment Score
