# Supplementary material for: Reactive oxygen species measure for rapid detection of infection in fluids
Source: Ann Intensive Care. 2016 Apr 29;6:41. doi: 10.1186/s13613-016-0142-8 (PMC4851674; doi:10.1186/s13613-016-0142-8)
Supplement: Supplementary file 1 — 10.1186/s13613-016-0142-8 Area under the curve of luminescence: HBSS and PMA. [file 13613_2016_142_MOESM1_ESM.docx]

**Figure S1: Area under the curve of luminescence: HBSS and PMA**


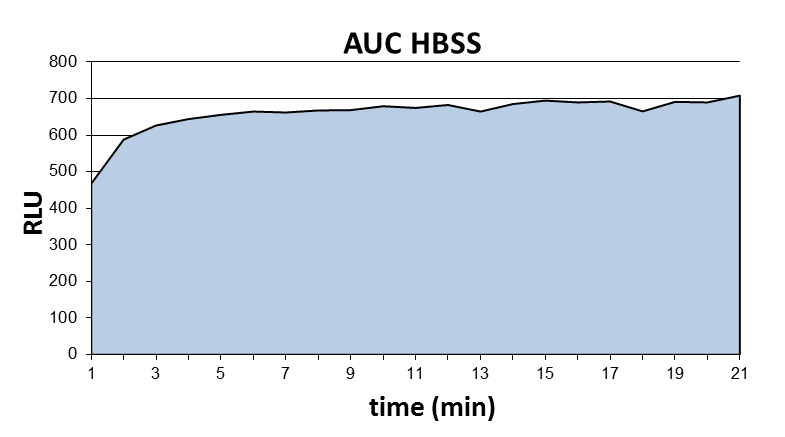

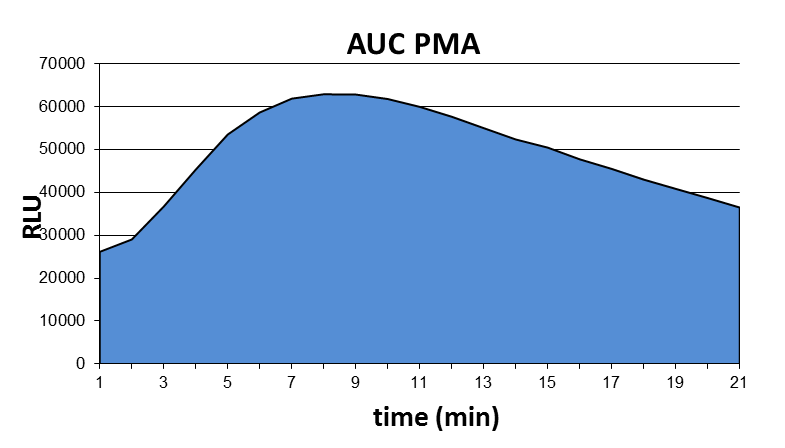


RLU: Relative Light Unit; AUC: Area Under the Curve; HBSS: Hanks’ Balanced Salt Solution; PMA: phorbol 12-myristate 13-acetate
