# Supplementary material for: Reactive oxygen species measure for rapid detection of infection in fluids
Source: Ann Intensive Care. 2016 Apr 29;6:41. doi: 10.1186/s13613-016-0142-8 (PMC4851674; doi:10.1186/s13613-016-0142-8)
Supplement: Supplementary file 2 — 10.1186/s13613-016-0142-8 ROS production in stimulated condition by PMA. [file 13613_2016_142_MOESM2_ESM.docx]

**Figure S2: ROS production in stimulated condition by PMA**


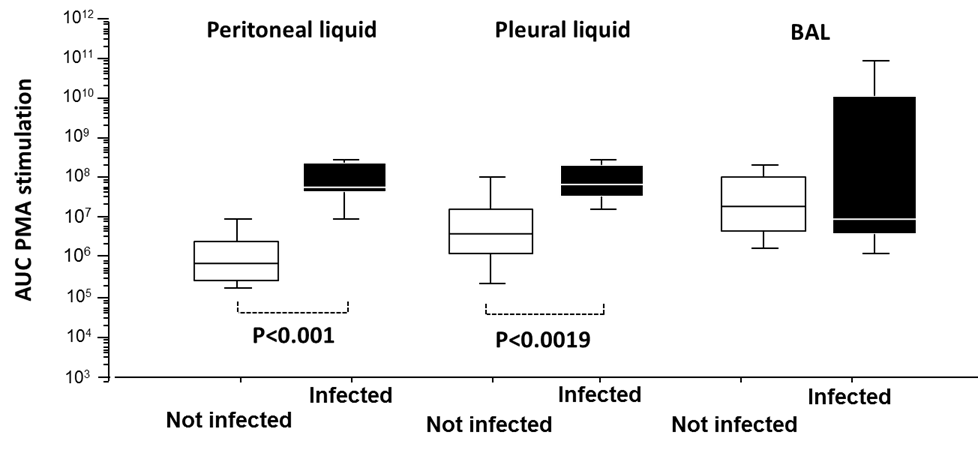


Area under the curve (AUC) of ROS production in stimulated condition by PMA. Number of peritoneal fluid (infected/non infected) n=17 (6/11), pleural fluid n = 28 (8/20), bronchoalveolar lavage (BAL) n = 20 (7/13).
