# Supplementary material for: Reactive oxygen species measure for rapid detection of infection in fluids
Source: Ann Intensive Care. 2016 Apr 29;6:41. doi: 10.1186/s13613-016-0142-8 (PMC4851674; doi:10.1186/s13613-016-0142-8)
Supplement: Supplementary file 3 — 10.1186/s13613-016-0142-8 Correlation tables between ROS production and PMN count in biological liquids. [file 13613_2016_142_MOESM3_ESM.docx]

**Figure S3: Correlation tables between ROS production and PMN count in biological liquids**


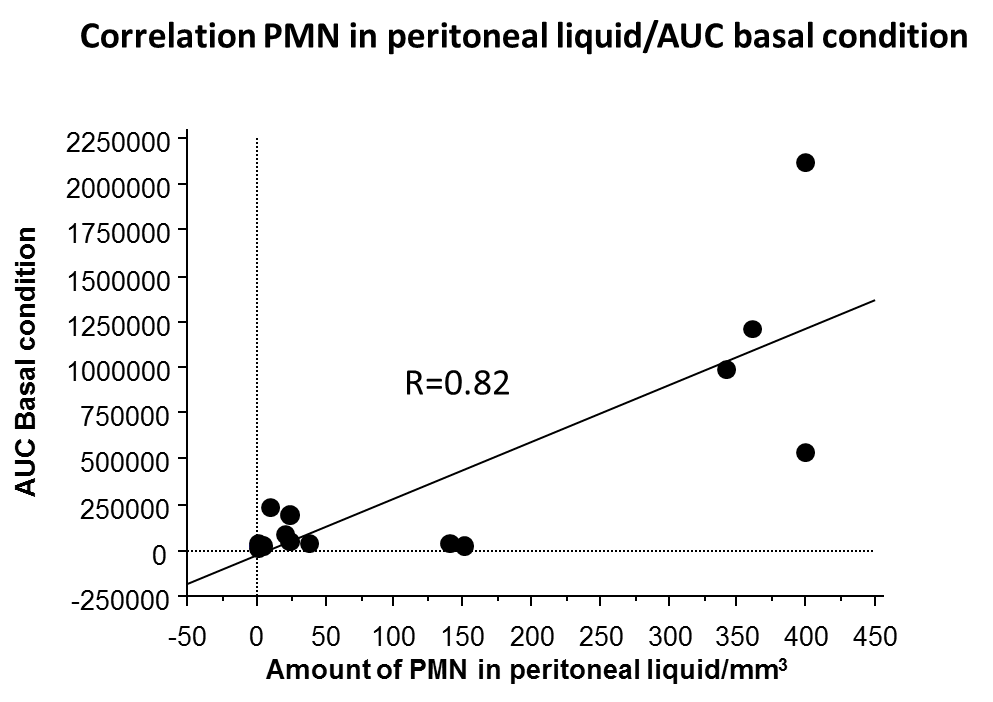
**A.**

**
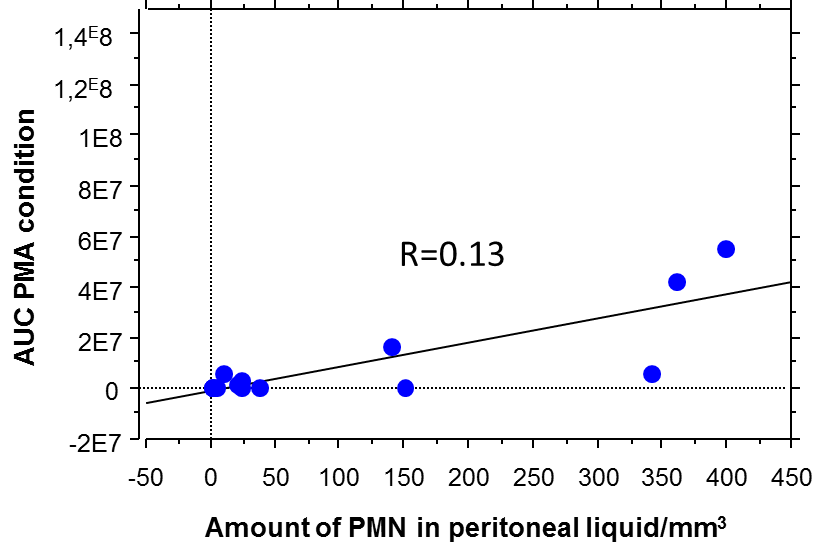
**

**Correlation PMN in peritoneal liquid/AUC PMA condition**

**B.**


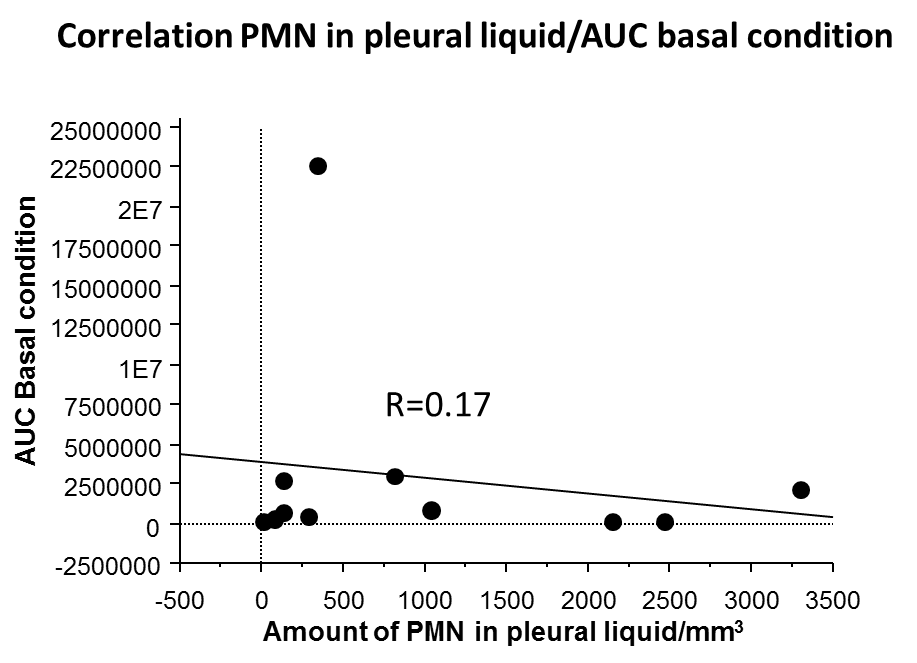


**Correlation PMN in Pleural liquid/AUC PMA condition**


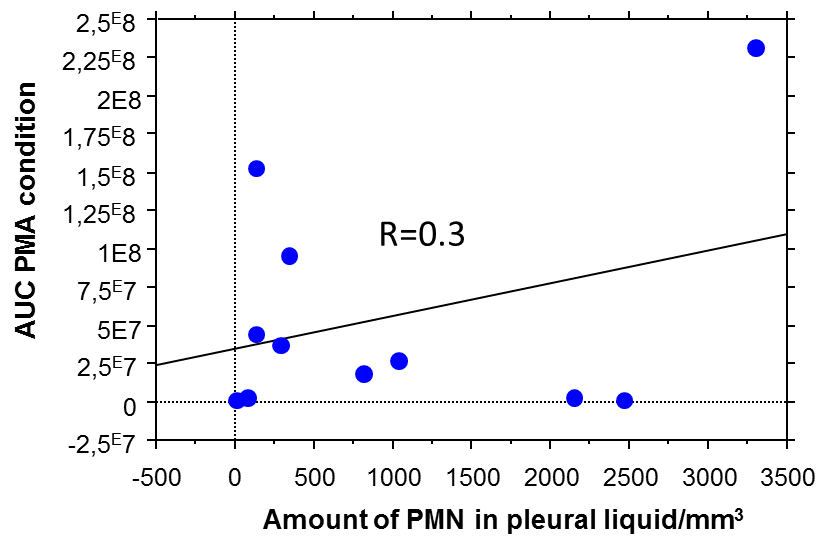


**
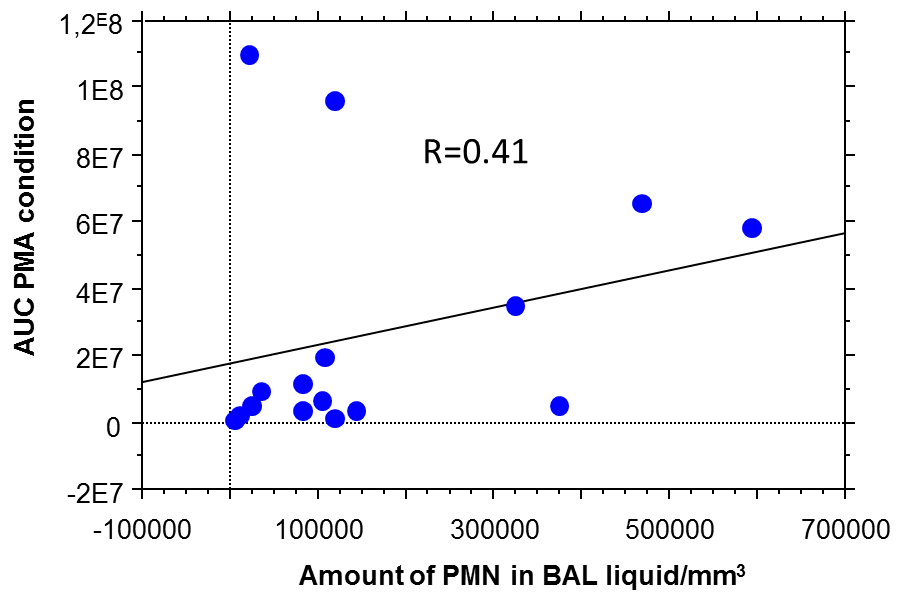
**
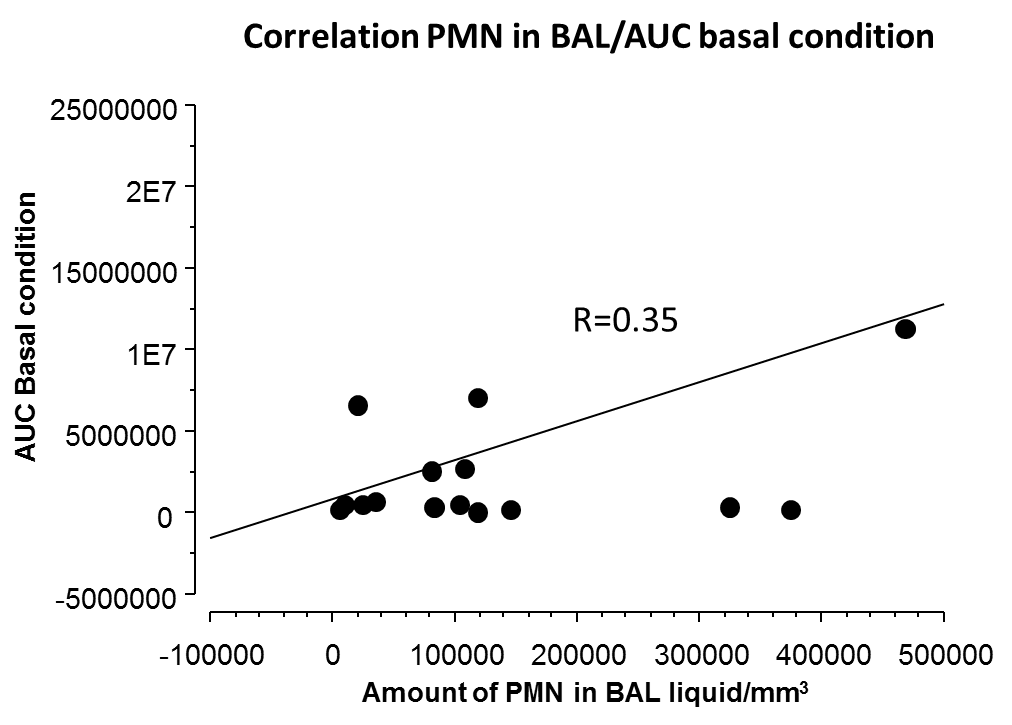
**C.**

**Correlation PMN in BAL/AUC PMA condition**

Correlation tables between the ROS production and the PMN count in A: peritoneal liquid, B: pleural liquid and C: bronchoalveolar lavage (BAL).

AUC: area under the curve; PMN: polymorphonuclear neutrophils; PMA: phorbol 12-myristate 13-acetate; R: correlation coefficient
